# Combining CRISPR-Cas12a-Based Technology and Metagenomics Next Generation Sequencing: A New Paradigm for Rapid and Full-Scale Detection of Microbes in Infectious Diabetic Foot Samples

**DOI:** 10.3389/fmicb.2021.742040

**Published:** 2021-10-07

**Authors:** Yixin Chen, Ya Shi, Weifen Zhu, Jiaxing You, Jie Yang, Yaping Xie, Hanxin Zhao, Hongye Li, Shunwu Fan, Lin Li, Chao Liu

**Affiliations:** ^1^Department of Endocrinology, Zhejiang University School of Medicine Sir Run Run Shaw Hospital, Hangzhou, China; ^2^Hangzhou Digital Micro Biotech Co., Ltd., Hangzhou, China; ^3^Department of Orthopedics, Zhejiang University School of Medicine Sir Run Run Shaw Hospital, Hangzhou, China; ^4^Department of Hematology, Affiliated Hangzhou First People’s Hospital, Zhejiang University School of Medicine, Hangzhou, China

**Keywords:** diabetic foot infections, *Staphylococcus aureus*, loop-mediated isothermal amplification, clustered regularly interspaced short palindromic repeats, metagenomics next generation sequencing

## Abstract

**Introduction:** Diabetic foot infections (DFIs) pose a huge challenge for clinicians. *Staphylococcus aureus*, including methicillin-resistant *S. aureus* (MRSA), is one of the most significant pathogens of DFI. Early pathogen identification will greatly benefit the diagnosis and treatment of the disease. However, existing diagnostic methods are not effective in early detection.

**Methods:** We developed an assay that coupled loop-mediated isothermal amplification (LAMP) and clustered regularly interspaced short palindromic repeats (CRISPR) techniques to enable quick and specific detection of *Staphylococcus aureus* and differentiate MRSA in samples from patients with DFI. Furthermore, the results were compared using a reference culture, quantitative real-time polymerase chain reaction (qRT-PCR), and metagenomics next generation sequencing (mNGS).

**Results:** The CRISPR-LAMP assay targeting *nuc* and *mecA* successfully detected *S. aureus* strains and differentiated MRSA. The limit of detection (LoD) of the real-time LAMP for *nuc* and *mecA* was 20 copies per microliter reaction in comparison to two copies per μL reaction for the qRT-PCR assay. The specificity of the LAMP-CRISPR assay for *nuc* was 100%, without cross-reactions with non-*S. aureus* strains. Evaluating assay performance with 18 samples from DFI patients showed that the assay had 94.4% agreement (17/18 samples) with clinical culture results. The results of mNGS for 8/18 samples were consistent with those of the reference culture and LAMP-CRISPR assay.

**Conclusion:** The findings suggest that the LAMP-CRISPR assay could be promising for the point-of-care detection of *S. aureus* and the differentiation of MRSA in clinical samples. Furthermore, combining the LAMP-CRISPR assay and mNGS provides an advanced platform for molecular pathogen diagnosis of DFI.

## Introduction

Diabetic foot ulcers, one of the most critical complications of diabetes mellitus, has become a significant public health problem. When patients with diabetic foot ulcers first consult physicians, over half of them have complications with diabetic foot infections (DFIs) (Prompers et al., 2006; Jia et al., 2017). Treatment of DFI requires precise evaluation of infection conditions, pathogen confirmation of infectious ulcers, appropriate selection of antibiotics, and surgical intervention, if necessary (Lipsky et al., 2012). DFI patients are often challenged by the complexity of intricate ulceration and moderate to severe infections, even sepsis. Broad-spectrum empirical antimicrobials would be the primary option for clinicians to hinder the further deterioration of infectious conditions and lower medical risks, which increases antibiotic exposure and selective pressure that contribute to the development of multi-drug resistant microorganisms. Nevertheless, a rapid, accessible, and accurate diagnostic test for pathogenic identification is valuable for clinical procedures.

*Staphylococcus aureus* is the dominant pathogen of DFI. The resistance rate of *S. aureus* to methicillin fluctuated between 15–30% (Eleftheriadou et al., 2010). It has been shown that infections caused by methicillin-resistant *S. aureus* (MRSA) are linked to higher mortality rates and greater consumption of medical resources than infections caused by methicillin-sensitive *S. aureus* (MSSA) (Cosgrove et al., 2003; Filice et al., 2010). Bacterial culture, quantitative real-time polymerase chain reaction (qRT-PCR) (Buchan et al., 2015; Ellem et al., 2015; Silbert et al., 2017), and metagenomics next generation sequencing (mNGS) (Charalampous et al., 2019; Kalan et al., 2019; Zou et al., 2020) are typically used to identify *S. aureus* and discriminate between MSSA and MRSA. Although traditional culture is the gold standard for pathogen diagnosis, it is time-consuming, and delayed results cannot provide timely clinical guidance. qRT-PCR has a shortened detection time; however, it has not been widely applied because of its high requirements for equipment and laboratory staff. mNGS, a novel detection method, is still not commonly accessible because of its high cost in terms of time, funding, instrumentation, and technical expertise.

In recent years, the application of isothermal amplification combined with clustered regularly interspaced short palindromic repeats (CRISPR) has been developed for pathogen diagnosis (Li et al., 2019). An innovative discovery reported that RNA-guided DNA binding activated Cas12a for both site-specific double-stranded DNA (dsDNA) *cis*-cleavage and indiscriminate single-stranded DNA (ssDNA) *trans*-cleavage. With the guidance of a single CRISPR RNA (crRNA), Cas12a recognizes a TTTN protospacer-adjacent motif (PAM) and binds to crRNA-complementary dsDNA (Li et al., 2018a,b). This indicates that the high specificity of the CRISPR/Cas12a assay is determined by PAM and crRNA. The coupling of isothermal amplification and *trans*-ssDNA repetitive cutting results in double-magnifying biosensing signals, indicating the high sensitivity of the assay. It has been proven to enable a rapid and accurate detection of the human papillomavirus and a new coronavirus (resulting in the disease referred to as COVID-19) in patient samples (Chen et al., 2018; Broughton et al., 2020).

In this study, we developed an assay coupling loop-mediated isothermal amplification (LAMP) and *trans*-cleavage of Cas12a for the quick and specific detection of *S. aureus* and the differentiation of MRSA in samples of patients with DFI. Furthermore, the results were compared with standard methods, including bacterial culture, qRT-PCR, and mNGS.

## Materials and Methods

### Nucleic Acid Preparation

DNA extraction from the tissues of DFI patients was performed using QuickExtract^TM^ DNA Extraction Solution (Lucigen, Beijing, China). Quick DNA extractions were finished after incubation at 65°C for 10 min and 98°C for 2 min. The sequences of *nuc* (*S. aureus*-specific gene) and *mecA* (encoding penicillin-binding protein-2a) were obtained from a public database. LAMP primers were designed against regions of *nuc* and *mecA* using PrimerExplorer v.5. with compatible gRNAs (Supplementary Table 1). The gRNA was designed with a TTTN structure (PAM) at the right end. The length of gRNA was 20-25bp, and the GC content was 40–60%. The design was assured to avoid the combination of gRNA and primer dimer (Table 1). The primers and gRNAs were obtained from Shanghai General Biotech Co., Ltd. (Shanghai, China).

**TABLE 1:** gRNA sequences for *nuc* and *mecA*.

gRNA	sequence (5′-3′)
nuc-gRNA	UAAUUUCUACUAAGUGUAGAUUACAUUAAUUUAACCGUAUCA
mecA-gRNA	UAAUUUCUACUAAGUGUAGAUUGCCAACCUUUACCAUCGAUU

### Real-Time Polymerase Chain Reaction Assay

A 20-μL system was used to conduct the real-time PCR assay for the detection of *nuc* and *mecA* with the following specifications: Forward primer for *nuc* gene: 5′-ACTGTAACTTTGGCACTGG-3′; Reverse primer for *nuc* gene: 5′-GCAGATACCTCATTACCTGC-3′; Probe: 5′-[6FAM]- ATCGCAACGACTGGCGCTA-[BHQ1]-3′ [12]; Forward primer for *mecA* gene: AAAACTAGGTGTTGGTGAAGATATACC; Reverse primer for *mecA* gene: GAAAGGATCTGTACTGGGTTAATCAG; Probe: [6FAM]-TTCACCTTGTCCGTAACCTGAATCAGCT-[BHQ1] (Sabet et al., 2007). Real-time PCR assays were performed using the Premix Ex Taq^TM^ (Probe qPCR) Kit (TaKara Biomedical Technology Co., Ltd., Beijing, China). Ten microliters Premix Ex Taq (Probe qPCR) (2×), 0.2 μM forward primer, 0.2 μM reverse primer, 0.1 μM probe, and 2 μL DNA templates of different concentrations were included in the reaction system. The temperature cycling conditions were 95°C for 30 s, followed by 40 cycles of 95°C for 5 s, and 60°C for 30 s. The reaction was performed using the StepOne^TM^Plus Real-Time PCR System (Applied Biosystems, Inc., Carlsbad, CA, United States).

### Real-Time Loop-Mediated Isothermal Amplification Assay

The real-time LAMP assay was conducted in a 25-μL mixture containing 1 × Bst DNA polymerase buffer (New England Biolabs, Beijing, China), 0.4 μM FIP primers, 0.4 μM BIP primers, 0.2 μM LF primers, 0.2 μM LB primers, 0.1 μM F3 primers F3, 0.1 μM B3 primers, 1.4 μM dNTPs (New England Biolabs), 8U Bst DNA polymerase (New England Biolabs), 8 mM MgSO_4_ (New England Biolabs), 1 × SYBR Green I (Thermo Fisher Scientific, Inc., Waltham, MA, United States), and 2 μL of DNA templates of different concentrations. Real-time LAMP assays of *nuc* and *mecA* were conducted in the same reaction system, except for the primers. The LAMP reaction solution was covered with 40 μL mineral oil. The reaction was performed at 65°C for 60 min in a StepOne^TM^ Plus Real-Time PCR System (Applied Biosystems, Inc., Carlsbad, CA, United States) and terminated at 80°C for 5 min. The fluorescence information was collected every 30 s during the reaction.

### Loop-Mediated Isothermal Amplification-Clustered Regularly Interspaced Short Palindromic Repeats Assay

The LAMP system contained the same mixture as the real-time LAMP assay. The fluorescent dyes were added into the system and the LAMP reagents covered with 40 μL of mineral oil. The LAMP reaction was followed by the admixture of CRISPR reaction solution (preloaded inside the lid) to the LAMP amplification solution by centrifugation. The 20 μL CRISPR reaction solution contained 1 × NEBuffer 2.1, reaction buffer, 0.2 μM EnGen^®^ Lba Cas12a (Cpf1) (New England Biolabs), 0.6 μM gRNA, 2.5 μM CRISPR probes, and 1 U/μL RNA inhibitor (Thermo Fisher Scientific, Inc., Waltham, MA, United States). Sequence information of gRNA is shown in Table 1. The CRISPR reaction was conducted in a ProFlex PCR System (Applied Biosystems, Inc., Carlsbad, CA, United States) at 37°C for 5 min. After the reaction, the visualized results were observed using a simple fluorescence reader.

### Sensitivity and Specificity of Loop-Mediated Isothermal Amplification-Clustered Regularly Interspaced Short Palindromic Repeats Assay

Amplification of *S. aureus* genomic DNA, including *nuc* and *mecA*, was performed using outer primers (F3 and B3, Supplementary Table 1). The lengths of the PCR products of *nuc* and *mecA* were 248 bp and 208 bp, respectively. The PCR product was purified using the TAKARA gel DNA fragment recovery kit, and the purified product was ligated to the pMD18-T vector (2692 bp). The recombinant plasmids were transformed into *Escherichia coli* DH5α competent cells. LB growth medium containing 10 μg/mL ampicillin was used to screen positive colonies. Plasmids of *E. coli* that comprised the pMD18-T vector and *nuc* (248 bp)/*mecA* (208 bp) were extracted using the TaKaRa MiniBEST Plasmid Purification Kit (TaKaRa, 9760). We then measured the concentration of plasmids and calculated the DNA copy number [6.02 × 10^23^ × concentration (ng/μL) × 10^9^/(DNA sequence length × 660)]. The concentration of plasmids containing *nuc* was 3.26 ng/μL. The DNA copy number of *nuc* was 1.0 × 10^9^ copies per μl, calculated according to the above formula. The concentration of plasmids containing *mecA* was 12 ng/μL, and the copy number was 3.78 × 10^9^ copies per μl. The plasmids were serially diluted to 1:10. Isothermal amplifications of the dilutions were repeatedly performed three times, and the limit of detection (LoD) was determined as the minimum DNA copy number of the diluted plasmids that could amplify the target genomic DNA.

Genomic DNA from 14 strains of *staphylococci* species was analyzed using the LAMP-CRISPR assay to examine the specificity of the LAMP assay. The strains were *S. epidermidis, S. hominis, S. cohnii, S. warneri, S. haemolyticus, S. aureus, S. capitis, S. saprophyticus, S. pasteuri, S. saprophyticus* ATCC^®^ 15305, *S. haemolyticus* ATCC ^®^29970, *S. epidermidis* ATCC ^®^14990, *S. caprae*, and *S. aureus* subsp. *sureus* ATCC ^®^12600.

### Metagenomic Next-Generation Sequencing

After DNA extractions using a DNeasy Blood and Tissue Kit (Qiagen, 69504, Shenzhen, China), DNA was fragmented using a Bioruptor Pico instrument to generate 200–300-bp fragments (Bioruptor Pico protocols). Then, libraries were constructed by MGIEasy (MGIEasy universal DNA library prep kit) as follows: first, the DNA fragments were subjected to end-repair sequencing, and A-tailing was added to one tube. Subsequently, the resulting DNA was ligated with bubble-adaptors that included barcode sequences and then amplified by the PCR method. Quality control was carried out using bioanalyzer (Agilent 2100, Agilent Technologies, Santa Clara, CA, United States) to assess DNA concentrations and fragment size. Qualified libraries were pooled together to make a single strand DNA circle (ssDNA circle), and then, DNA nanoballs (DNB) were generated by rolling circle replication (RCA). The final DNBs were loaded into the sequence chip and sequenced on the BGISEQ platform using 50 bp/single-end sequencing. Finally, optical signals were collected using a high-resolution imaging system, and then, these signals were transformed into digital information, which can be decoded into DNA sequence information.

### Reference Culture Method and Antimicrobial Susceptibility Test

The sample culture broths were plated on blood agar and incubated for 18–48 h. Identification of S. aureus and examination of antimicrobial susceptibility were conducted using a fully automatic vitek-2 compact system (Biomerieux, Shanghai, China). The results were interpreted on the basis of the guidelines (M100, 28th edition) established by the Clinical and Laboratory Standards Institute (CLSI, 2018).

### Human Clinical Sample Collection

Tissues of infected ulcers were acquired from DFI patients at their first visit to the outpatient clinic or the Department of Emergency at Sir Run Run Shaw Hospital. After the wound was cleaned with saline solution, DFU tissue with the size of a rice grain was taken out with a blade and immediately sent to the laboratory for DNA extraction. The study was approved by the Human Research Ethics Committee of Sir Run Run Shaw Hospital.

### Statistical Methods

All reactions were repeated three times, the CT value of the real-time PCR is shown as “mean ± standard deviation” (x ± s).

## Results

### Real-Time Loop-Mediated Isothermal Amplification Assay for nuc and mecA

**FIGURE 1:**
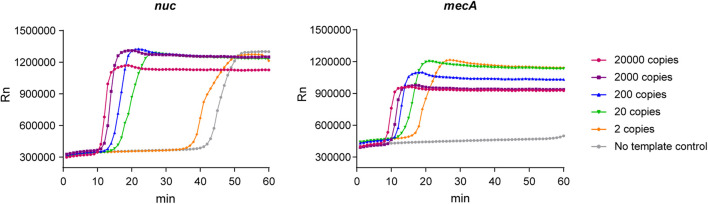
Amplification curves of the real-time LAMP assay for *nuc* and *mecA*.

We conducted a real-time LAMP assay for amplifying nuc and mecA with seven dilutions. The amplification curves of the dilutions with serial concentrations are shown in Figure 1. The LAMP assay was able to amplify genomic DNA targets. There was no stable fluorescent signal obtained when the genomic DNA template was at two copies per μL reaction.

### Visual Detection Assay With Clustered Regularly Interspaced Short Palindromic Repeats/Cas12a

**FIGURE 2:**
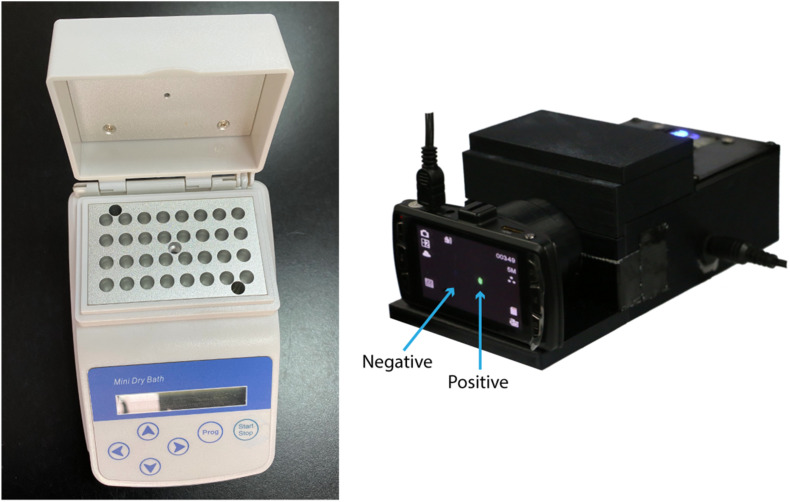
Portable equipment for the LAMP assay and fluorescence visualization.

**FIGURE 3:**
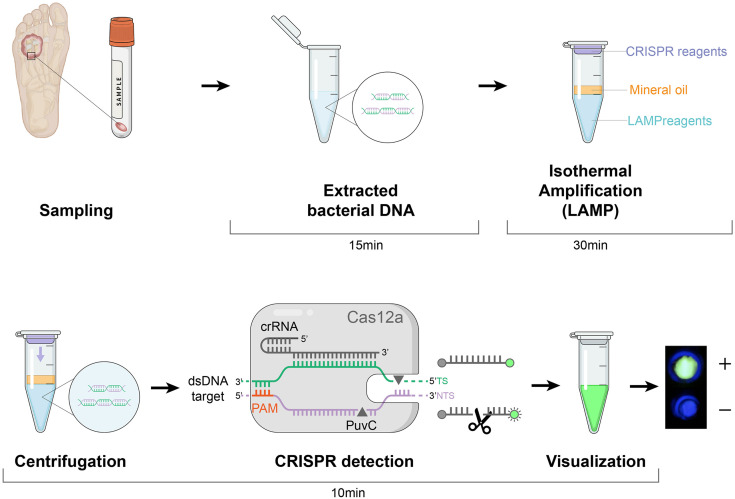
Schematic of the LAMP-CRISPR assay workflow. Quick DNA extraction was used as an input to the LAMP-CRISPR system (LAMP preamplification and Cas12-based detection for *nuc* and *mecA*). The result is visualized by a fluorescence reader.

**FIGURE 4:**
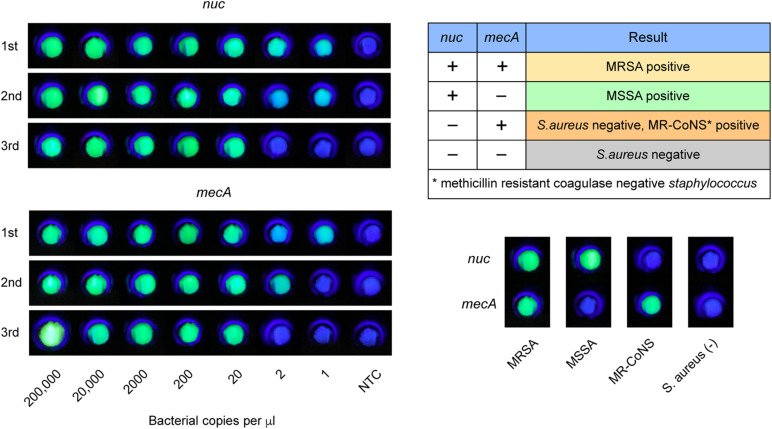
LAMP-CRISPR assay readout. Fluorescent results of MRSA, MSSA, MR-CoNS, and *S. aureus* are presented at the bottom right. *Nuc* detection indicates *S. aureus* positive. MRSA identification requires positive for both *nuc* and *mecA*.

Next, we used the CRISPR/Cas12a system to realize the visual readout of the targeted amplicons, based on the collateral DNA cleavage activity of the Cas12a effector. The cleaved ssDNA reporters produced fluorescent signals, which could be seen through a portable fluorescence reader (Figure 2). The entire procedure and explanation of the results are presented in Figure 3. Pre-loading of the CRISPR reagents inside the lid avoided the need to open the cap during the process. We performed this assay for detecting *nuc* and *mecA* with serial dilutions. Green fluorescence was observed when the DNA sample was 2 × 10^5^, 2 × 10^4^, 2 × 10^3^, 2 × 10^2^, and 2 × 10^1^ copies, which was referred to as a positive result. No green fluorescence signal was seen for a sample of 2 × 10^0^ copies, one copy, and the no template control (NTC) (Figure 4). The interpretation of the genetic results for bacterial identification are illustrated in Figure 4.

### Sensitivity of the Loop-Mediated Isothermal Amplification-Clustered Regularly Interspaced Short Palindromic Repeats Assay

**FIGURE 5:**
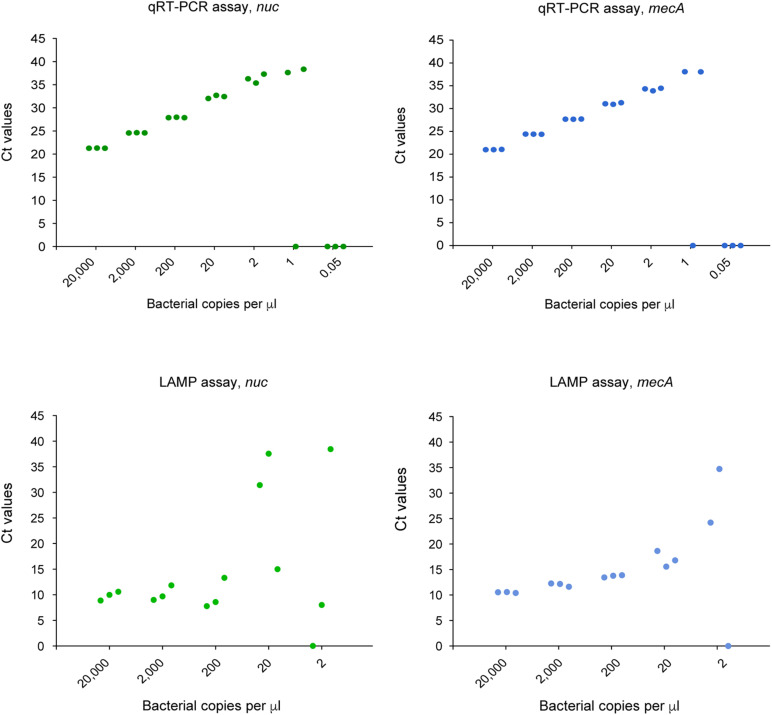
LoD of the qRT-PCR and CRISPR-LAMP assay for *nuc* and *mecA*.

We compared the LoD of the LAMP assay relative to the qRT- PCR assay for the detection of *S. aureus* and MRSA. A standard scatter plot was plotted using seven serial dilutions with three replicates at each dilution (Figure 5). The LoD of the LAMP assay for *nuc* was 20 copies per μL reaction in comparison to two copies per μL reaction for the qRT-PCR assay. For *mecA*, the LoD of the LAMP assay was 20 copies per μL reaction, versus two copies per μL reaction for the qRT-PCR assay.

### Specificity of the Loop-Mediated Isothermal Amplification-Clustered Regularly Interspaced Short Palindromic Repeats Assay

**FIGURE 6:**
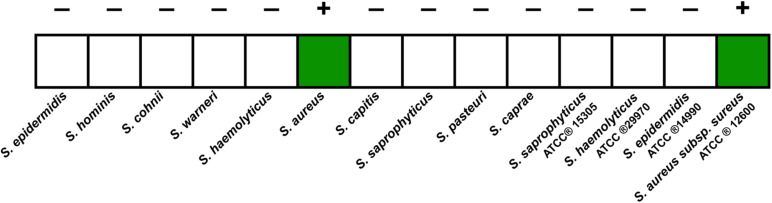
Results of the LAMP-CRISPR assay for 14 strains of *staphylococci* species.

We tested the extracted DNA templates from strains of several staphylococcus species using the LAMP-CRISPR assay with primers for *nuc*. These strains included ten isolates, including *S. epidermidis*, *S. hominis*, *S. cohnii*, *S. warneri*, *S. haemolyticus*, *S. aureus*, *S. capitis*, *S. saprophyticus*, *S. pasteuri*, and *S. caprae*, and four purchased strains, including *S. saprophyticus* ATCC^®^ 15305, *S. haemolyticus* ATCC ^®^29970, *S. epidermidis* ATCC ^®^14990, and *S. aureus* subsp. *aureus* ATCC ^®^12600. Positive results were generated only for DNA samples from *S. aureus* (Figure 6). It was confirmed that the LAMP-CRISPR assay for *nuc* could specifically detect *S. aureus*, with no cross-reactions to other staphylococcal families.

### Application of the Loop-Mediated Isothermal Amplification-Clustered Regularly Interspaced Short Palindromic Repeats Assay to the Diabetic Foot Infection Patient Samples

Clinical validation of this assay was performed using 18 samples of isolated strains from DFI ulcers. Confirmatory qRT-PCR was used to establish target gene detected by the LAMP-CRISPR assay. The results of the two molecular methods were compared to those of the gold-standard culture method (Table 2). For *S. aureus* detection, six samples were *nuc-*positive as per the CRISPR-LAMP assay and *S. aureus-*positive by culture. For MRSA detection, two samples were positive for both *nuc* and *mecA* as per the CRISPR-LAMP assay and MRSA-positive by culture. One discordant sample (No. 8) was positive for both *nuc* and *mecA* as per the LAMP-CRISPR assay and MRSA-negative by culture. The results showed that MSSA was mixed with methicillin-resistant *Staphylococcus pippi*.

**TABLE 2:** Results of 18 samples from DFI patients using the LAMP-CRISPR assay, real-time PCR, and the reference culture.

Patient	LAMP-CRISPR		Real-time PCR		Culture
	*nuc*	*mecA*	*nuc*	*mecA*	
1	+	–	+(38.337)	–	MSSA
2	–	–	–	–	*Proteus vulgaris*
3	–	–	–	–	*Klebsiella pneumoniae*
4	–	–	–	37.454	*Klebsiella pneumoniae*
5	+	+	+(26.836)	+ (27.123)	MRSA
6	–	–	–	–	*Acinetobacter baumannii*
7	–	–	–	–	*Staphylococcus hominis*
8	+	+	+(35.05)	+ (31.203)	MSSA,Staphylococcus pippi*
9	–	–	–	–	*Pseudomonas aeruginosa*
10	–	–	–	–	*Proteus vulgaris*
11	+	–	+(24.945)	–	MSSA
12	–	–	–	–	*Proteus vulgaris*
13	+	–	+(28.193)	–	MSSA
14	–	–	–	37.217	*Morganella morganii*
15	+	+	+(30.227)	+ (29.936)	MRSA
16	–	–	–	–	*Enterococcus faecalis*
17	–	–	–	–	*Staphylococcus epidermidis*
18	–	–	–	–	*Proteus mirabilis*

*Methicillin resistant-coagulase negative staphylococcus.

### Metagenomics Next Generation Sequencing Analysis

**FIGURE 7:**
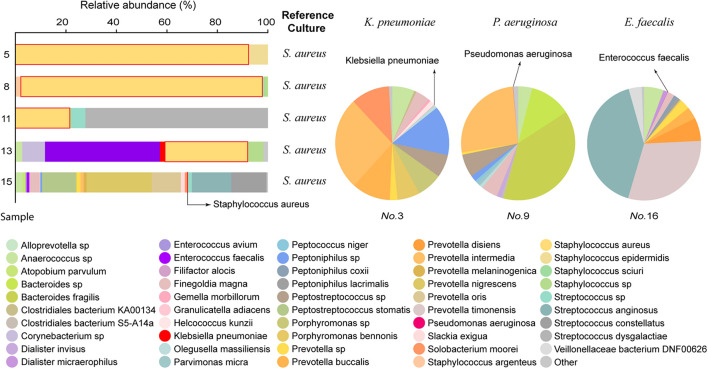
Comparison of mNGS data to the reference culture. In the left 100% stacked columns, the column representing *S. aureus* is framed by a red line.

After LAMP-CRISPR and qRT-PCR analysis, 8/18 samples were selected for mNGS. The culture growth of these eight samples indicated pathogens commonly seen in clinical practice, such as *S. aureus*, *Enterococcus faecalis*, *Pseudomonas aeruginosa*, *and Klebsiella pneumoniae*. The results of the mNGS analysis are listed in Supplementary Material (Supplementary Table 2). The mNGS results were consistent with the reference culture (Figure 7).

## Discussion

Here, we combined the CRISPR-Cas12a-based technique with LAMP to develop a rapid diagnostic assay for the detection of *S. aureus* in samples directly from patients with DFI. The CRISPR-LAMP assay targeting *nuc* and *mecA* successfully identified *S. aureus* strains and differentiated MRSA from MSSA. The entire procedure was brief and contamination-free. Visualization of the results through a simple device also made them easy to interpret. This molecular assay could be promising for the point-of-care detection of *S. aureus* from clinical samples at some underprovided hospitals and areas.

MRSA can be transmitted by contact between the medical staff and patients. Infection-control precautions, such as the primary isolation of patients with MRSA infection and disinfection of the clinic environment after every diabetic foot patient with MRSA infection, can prevent cross-transmission of MRSA (Lecornet et al., 2007). Early detection of MRSA can alert hospital staff to carry out the necessary precautions to reduce the spread of MRSA among patients. With the worldwide prevalence of MRSA isolates and frequent exposure to vancomycin, the appearance of vancomycin-intermediate *S. aureus* (Centers for Disease Control and Prevention (CDC), 2002; Chang et al., 2003) as well as vancomycin-resistant *S. aureus* (Centers for Disease Control and Prevention (CDC), 1997) strains have been identified globally. Early discrimination between MSSA and MRSA could reduce the inappropriate use of broad-spectrum antimicrobials and decrease the selective pressure that initiates the emergence and spread of resistance in bacteria (Gardete and Tomasz, 2014).

A potential weakness of this assay was the partial disagreement between the phenotype and genotype of microorganisms, which was also observed in other molecular strategies such as GeneOhm MRSA or Xpert MRSA assays (Buchan et al., 2015). Microbes that were *mecA-*negative might contain *mecC*, which was recently discovered and identified as a novel resistance gene in MRSA (Lakhundi and Zhang, 2018). DFI is often caused by multiple bacteria (Lipsky et al., 2012), which indicates that the culture growth of a clinical sample could be a mixture of *S. aureus* and coagulase-negative *Staphylococcus*, both of which can carry the *mecA* gene. Therefore, apart from being caused MRSA alone, the double positivity of *nuc* and *mecA* could be explained by mixed Staphylococcal isolates. This discrepancy was observed in one patient (No. 8). Using long-range PCR may encompass the amplicons, including both *nuc* and *mecA*, from a single strain that can uniquely identify MRSA (McClure et al., 2020). This will compensate for the current deficiency of the LAMP or regular PCR assays and improve specificity.

**FIGURE 8:**
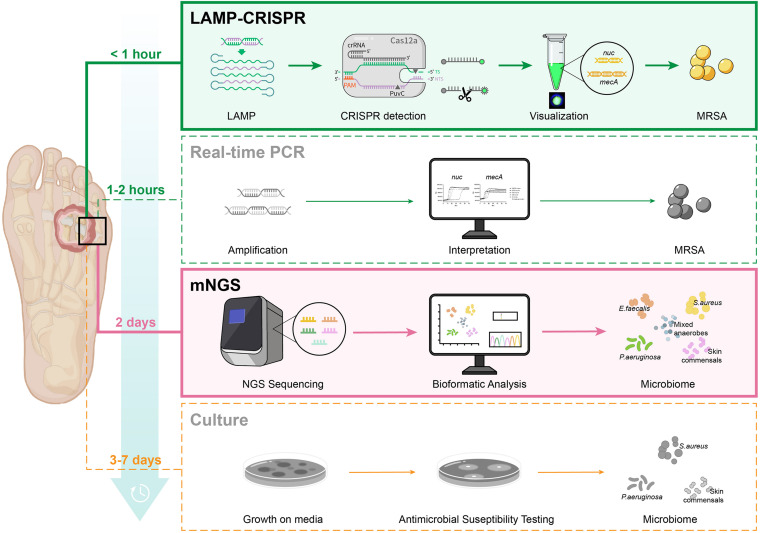
Procedure of four diagnostic methods: LAMP-CRISPR assay, real-time PCR, mNGS, and reference culture.

We then compared this novel LAMP-CRISPR assay with the reference culture, using qRT-PCR and mNGS methods (Figure 8). Compared to other diagnostic technologies, the CRISPR-LAMP assay enabled the detection of targeted genes at very low DNA concentrations within 1 h, which is faster than qRT-PCR and no complex infrastructure was required (Table 3). DFI is a rapidly progressing and relatively critical disease that may lead to disability and mortality. CRISPR-LAMP detection could aid clinicians in selecting targeted antibiotics and prevent the spread of multi-drug resistant microbes. Application of the CRISPR-LAMP assay optimizes the early treatment of DFI and complements the current paradigm for clinical diagnostics.

**TABLE 3:** Comparison of the LAMP-CRISPR assay to real-time PCR, mNGS, and the reference culture.

	LAMP-CRISPR	Real-time PCR	mNGS	Culture
Assay sample-to-result time	<1 h	1–2 h	2 days	3–7 days
Sensitivity	High	High	High	Low
Specificity	High	High	Low	High
complex laboratory infrastructure required	No	Yes	Yes	Yes
Cost	Inexpensive	Inexpensive	Expensive	Inexpensive

We also chose several representative samples for mNGS. mNGS can unbiasedly identify multiple microbes and address the limitations in pathogen width. It provides comprehensive information on the microbial profile of DFIs, including some rare pathogens that cannot be detected by conventional methods. mNGS can also provide quantitative data on the microbial concentration in the sample by counting sequenced reads, which is useful for polymicrobial samples (Wei et al., 2019). Study of the microbiome can potentially improve the diagnosis and treatment of DFIs. This revolutionary genetic approach further advances the management procedure of DFIs, which is becoming a rising trend in the clinical detection of infectious diseases. With the rapid determination of pathogens via CRISPR-LAMP and comprehensive supplementation of the bacterial spectrum by mNGS, a combination of these two methods can realize a timely and accurate pathogenic diagnosis of DFIs.

## Conclusion

Targeting both *nuc* and *mecA*, our LAMP-CRISPR assay has proven to be a rapid (within 1 h) and effective method for detecting *S. aureus* and distinguishing MRSA from MSSA in DFI samples. Due to its convenient visualized readout and portable nature, this method is promising for improving point-of-care detection. mNGS can provide comprehensive information for the microbiome study of DFI samples. Combining the LAMP-CRISPR technique and mNGS expanded and improved the pathogen diagnostic routine of DFI.

## Data Availability Statement

The data presented in this study are deposited in the EBI Metagenomics repository, accession numbers ERR6769676–ERR6769683.

## Ethics Statement

The studies involving human participants were reviewed and approved by Human Research Ethics Committee of Sir Run Run Shaw Hospital. The patients/participants provided their written informed consent to participate in this study.

## Author Contributions

CL, LL, and YC conceived and designed the study. YC was the major contributor in manuscript writing. YS, YC, and WZ completed the laboratory procedures. JaY, JeY, HL, and SF collected the samples of DFI patients and managed the diagnosis and treatment of patients. HZ analyzed the mNGS Data. YX proofread the first draft of the manuscript. CL and LL reviewed the manuscript. All authors agree to be accountable for the content of the work.

## Conflict of Interest

YS was employed by Hangzhou Digital-Micro Biotech Co., Ltd. The remaining authors declare that the research was conducted in the absence of any commercial or financial relationships that could be construed as a potential conflict of interest.

## Publisher’s Note

All claims expressed in this article are solely those of the authors and do not necessarily represent those of their affiliated organizations, or those of the publisher, the editors and the reviewers. Any product that may be evaluated in this article, or claim that may be made by its manufacturer, is not guaranteed or endorsed by the publisher.

## Supplementary Material

Supplementary Table 1 The sequences of the LAMP primers for *nuc* and *mecA*, and the CRISPR probe.

Supplementary Table 2 The results of the mNGS analysis. The highlights in the table represent the consistent microbials detected both by the mNGS and reference culture.

## Abbreviations

DFI: diabetic foot infections
MRSA: methicillin-resistant *Staphylococcus aureus*
MSSA: methicillin-sensitive *Staphylococcus aureus*
LAMP: loop-mediated isothermal amplification
CRISPR: clustered regularly interspaced short palindromic repeats
mNGS: metagenomics next generation sequencing
qRT-PCR: quantitative real-time polymerase chain reaction
ssDNA: single-stranded DNA
dsDNA: double-stranded DNA
crRNA: CRISPR RNA
PAM: protospacer-adjacent motif
LoD: limit of detection
DNB: DNA nanoballs
RCA: rolling circle replication
NTC: no template control.
